# Optimum Planting Density Improves Resource Use Efficiency and Yield Stability of Rainfed Maize in Semiarid Climate

**DOI:** 10.3389/fpls.2021.752606

**Published:** 2021-11-12

**Authors:** Yuanhong Zhang, Zonggui Xu, Jun Li, Rui Wang

**Affiliations:** ^1^College of Agronomy, Northwest A&F University, Yangling, China; ^2^Key Laboratory of Crop Physi-ecology and Tillage Science in Northwestern Loess Plateau, Ministry of Agriculture, Yangling, China; ^3^College of Forestry, Northwest A&F University, Yangling, China

**Keywords:** density, resources use efficiency, photosynthetic characteristic, rainfed maize, grain yield

## Abstract

Increasing planting density is an effective strategy for improving maize productivity, but grain yield does not increase linearly with the increase in plant density, especially in semiarid environments. However, how planting density regulates the integrated utilization of key input resources (i.e., radiation, water, and nutrients) to affect maize production is not clear. To evaluate the effects of planting density and cultivar on maize canopy structure, photosynthetic characteristics, yield, and resource use efficiency, we conducted a successive field experiment from 2013 to 2018 in Heyang County (Shaanxi Province, China) using three different cultivars [i.e., Yuyu22 (C1), Zhengdan958 (C2), and Xianyu335 (C3)] at four planting densities [i.e., 52,500 (D1), 67,500 (D2), 82,500 (D3), and 97,500 (D4) plants ha^–1^]. Increasing planting density significantly increased the leaf area index (LAI) and the amount of intercepted photosynthetically active radiation (IPAR), thereby promoting plant growth and crop productivity. However, increased planting density reduced plant photosynthetic capacity [net photosynthetic rate (Pn)], stomatal conductance (Gc), and leaf chlorophyll content. These alterations constitute key mechanisms underlying the decline in crop productivity and yield stability at high planting density. Although improved planting density increased IPAR, it did not promote higher resource use efficiency. Compared with the D1 treatment, the grain yield, precipitation use efficiency (PUE), radiation use efficiency (RUE), and nitrogen use efficiency (NUE) increased by 5.6–12.5%, 2.8–7.1%, and −2.1 to 1.6% in D2, D3, and D4 treatments, respectively. These showed that pursuing too high planting density is not a desirable strategy in the rainfed farming system of semiarid environments. In addition, density-tolerant cultivars (C2 and C3) showed better canopy structure and photosynthetic capacity and recorded higher yield stability and resource use efficiency. Together, these results suggest that growing density-tolerant cultivars at moderate planting density could serve as a promising approach for stabilizing grain yield and realizing the sustainable development of agriculture in semiarid regions.

## Introduction

Rainfed farming is a main agricultural production system practiced on more than 70% of the arable land in the world and accounts for approximately 60–65% of the global grain production (Lin and Liu, 2016). Therefore, it is important to ensure food security and increasing the economic status of local populations in the face of climate change. The Loess Plateau region, a typical intensive agroecosystem that covers a total area of 630,000 km^2^ in northwest China, has become an important cereal crop production belt (Zhang et al., 2014). This area has a long history of agricultural cultivation, and maize is one of the most important crops grown in this region. However, due to water scarcity, this area has always been dominated by dryland farming. Rainfall, which is the main resource for crop growth in this region, shows large inter- and intra-annual variability (Zhang et al., 2017), leading to low and unstable crop productivity. However, this region receives an ample amount of sunlight, which provides the energy required for obtaining a high yield (Teixeira et al., 2014). Therefore, to establish sustainable agriculture in this region, it is important to determine how the limited resources can be effectively utilized for improving crop yield and resource (i.e., radiation, water, and nutrient) use efficiency and for stabilizing crop productivity.

In maize (*Zea mays* L.), increasing planting density has proven to be an effective agronomic practice for improving grain yield and resource use efficiency worldwide (Testa et al., 2016; Jia et al., 2018; Fahad et al., 2020). However, only a few studies have explored how changes in the absorption and utilization of radiation, nutrients, and water caused by increasing planting density improve crop growth, development, and grain yield. Planting density affects the absorption and utilization of radiation, water, and nutrients in plants by changing the canopy and/or root system architecture (Hammer et al., 2009; Du et al., 2021). Increased planting density improves the intercepted photosynthetically active radiation (IPAR) by rapid canopy closure and increases the leaf area index (LAI) (Teixeira et al., 2014; Hernández et al., 2020). It is well-known that biomass yield is the production of IPAR, which ultimately converts into yield, and maize grain yield is determined by the product of total biomass (Du et al., 2021). Increasing planting density increases IPAR, but it also increases competition among plants for light, water, and nutrients (Ciampitti and Vyn, 2011; Rossini et al., 2011), causing abiotic stress in plants, which is often visually apparent in maize *via* the reduction in leaf area, leaf chlorophyll content, and grain biomass (Osakabe et al., 2014). Such phenomena decrease plant light interception and photoassimilate production, thereby decreasing crop productivity and resource use efficiency (Teixeira et al., 2014; Zhang et al., 2019b; Du et al., 2021). Under abiotic stress conditions, dry matter allocation to reproductive organs declines, leading to lower grain yield, yield components (i.e., kernel number and weight), and harvest index (HI) (Ciampitti and Vyn, 2011; Mylonas et al., 2020). Different cultivars also show different responses to planting density in terms of productivity and resource utilization efficiency (Balkcom et al., 2011; Tokatlidis et al., 2011; Tokatlidis, 2013). Therefore, it is important to understand how crop production and resource use efficiency respond to both planting density and plant genotype. In contrast, interactions within the above physiological indexes have also been recorded (Ciampitti and Vyn, 2012), and the enhanced knowledge of physiological relationships can be useful for developing maize management systems that improve resource use efficiency.

In this study, we conducted a 6-year successive field experiment on maize in the Loess Plateau region to (1) investigate the effects of planting density and cultivar on canopy structural characteristics, (2) explore the effects of planting density on plant growth and photosynthetic characteristics, and (3) evaluate the yield stability and resource (i.e., radiation, nitrogen, and water) use efficiency of dryland maize under different treatments.

## Materials and Methods

### Field Management and Experimental Design

Successive field experiments were conducted from 2013 to 2018 at the experimental station of the Heyang Dryland Agricultural Research Station of Northwest A & F University, located in the Heyang County of Shaanxi Province (35°19’ N, 110°4’ E, and 877 m above sea level), in the southeast region of the Loess Plateau in northwest China. At the experimental site, the average annual precipitation is approximately 494 mm (2004–2017), with approximately 60% of the annual rainfall occurring in July–September. The soil type is dark loessial soil and is classified as middle loam soil, according to the FAO/UNESCO Soil Classification (1993).

The experiment was arranged in a split-plot design with three replications. Planting density was assigned to the main plots, and maize cultivar was assigned to subplots. Four planting densities were evaluated in the experiment as follows: 52,500 plants ha^–1^ (D1), 67,500 plants ha^–1^ (D2), 82,500 plants ha^–1^ (D3), and 97,500 plants ha^–1^ (D4), with a row-to-row spacing of 50 cm. Three cultivars with different levels of tolerance to planting density were used in the experiment as follows: Yuyu22 (C1), Zhengdan958 (C2), and Xianyu335 (C3) (Xue et al., 2010). Other field management practices followed in this study have been described previously (Zhang et al., 2019c).

### Weather-Related Data

**FIGURE 1:**
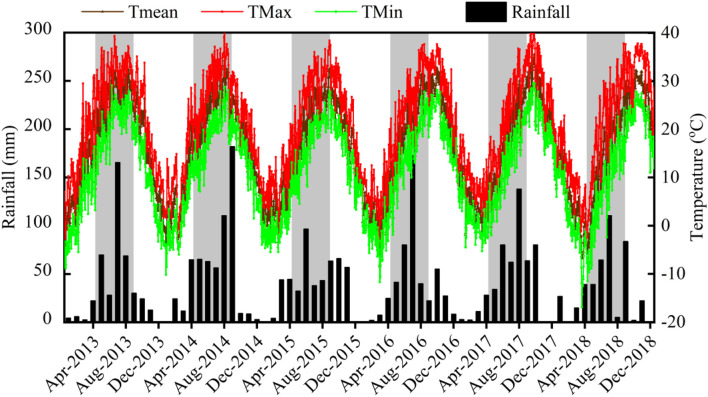
Dynamics of temperature and rainfall during the experimental period. The gray areas represent the growing period of maize.

Daily weather datasets (i.e., solar radiation, air temperature, and rainfall) were obtained from the national meteorological database,^1^ and the data from 2013 to 2018 are shown in Figure 1.

### Leaf Area Index and Aboveground Biomass

Five plants were randomly selected at different stages to determine the green leaf area (leaf length × leaf width × 0.75) and LAI (total leaf area per ha) of each maize plant (Zhang et al., 2019b). After measuring leaf area, the same plants were used for measuring the aboveground biomass. To measure the aboveground biomass, plants were fixated at 105°C for 0.5 h and then oven-dried at 85°C for a minimum of 48 h until a constant weight was achieved.

### Leaf Photosynthetic Characteristics and Chlorophyll Content

Five plants were randomly selected from each plot at the jointing (V6), tasseling (VT), and filling (R3) stages, and the net photosynthetic rate (Pn), transpiration rate (Tr), and stomatal conductance (Gc) of leaves were measured using a Li-6400 portable photosynthesis system (Li-COR Inc., Lincoln, NE, United States). These measurements were taken between 9:00 a.m. and 11:00 a.m. on a clear sunny day. The largest leaf was sampled at the V6 stage, while the maize ear leaf was sampled at the VT and R3 stages. Leaf chlorophyll content was determined using photometric methods, as described by Cui et al. (2019).

### Intercepted Photosynthetically Active Radiation and Radiation Use Efficiency

The IPAR (MJ m^–2^) per plant canopy and radiation use efficiency (RUE) (g MJ^–1^) data were determined using the following equations (Zhang et al., 2019b):


$$IPAR=\sum 0.5R\left(1-e^{-kLAI}\right)$$

$$RUE=\frac{\mathrm{Grain}\mathrm{yield}}{\text{IPAR}}$$

where *R* is the daily solar radiation (MJ m^–2^ day^–1^), *k* is the light extinction coefficient (0.65 for maize), and LAI is the LAI.

### Grain Yield

In each treatment, three random quadrats covering a 9.0 m^2^ area were selected to determine yield and yield components (kernel number per square meter and 100-kernel weight). Grain and biomass yield were determined at 14% moisture content. HI and precipitation use efficiency (PUE) were calculated using the following equations:


$$HI=\frac{Grainyield}{Biomassyield}$$

$$PUE=\frac{Grainyield}{P}$$

where *P* is the amount of precipitation (mm) during the growing season.

Crop yield stability, as affected by different treatments, was evaluated based on its variability by measuring the coefficient of variation (CV, %) using the following equation (Xu et al., 2019):


$$CV=\frac{STD\left(Yt\right)}{AVE\left(Yt\right)}\times 100$$

where *STD(Yt)* is the SD of grain yield of a particular treatment over the 6-year experiment period, and *AVE(Yt)* is the mean yield of that treatment over the same period.

The sustainable yield index (SYI) is a quantitative measure to assess the sustainability of any agricultural system (Sharma et al., 2013). The SYI was calculated using the following equation (Li et al., 2016):


$$SYI=\frac{AVE\left(Yt\right)-STD\left(Yt\right)}{Ymax}$$

where *Ymax* represents the maximum crop yield attained by any treatment during the study period, and *AVE(Yt)* is the mean yield of that treatment over the same period.

### Nitrogen Uptake and Utilization

The sampled maize plants were separated into different organs. Samples were then oven-dried at 85°C to measure the dry matter weight. Nitrogen concentration in plant samples was analyzed based on the Kjeldahl method (Du et al., 2021). Nitrogen uptake, nitrogen harvest index (NHI), nitrogen use efficiency (NUE), nitrogen productive efficiency (NPE), and nitrogen uptake efficiency (NUPE) were calculated as follows (Zhang et al., 2019b):


$$NUE=\frac{Grainyield}{Totalnitrogenuptake}$$

$$NUPE=\frac{Totalnitrogenuptake}{Nitrogenapplicationrate}$$

$$NPE=\frac{Grainyield}{Nitrogenapplicationrate}$$

$$NHI=\frac{Grainnitrogenuptake}{Totalnitrogenuptake}$$

### Statistical Analysis

The statistical significance of density, cultivar, and their interaction was assessed with two-way ANOVA. All data were analyzed using the IBM SPSS statistical software package (version 20.0, SPSS Inc., Chicago, IL, United States), followed by the least significant difference (LSD) test. Differences among treatments were considered statistically significant at *p* < 0.05, and figures were generated using Origin 2015 (v. Pro 2019; OriginLab Corp., Northampton, MA, United States).

## Results

### Biomass and Grain Yield

**FIGURE 2:**
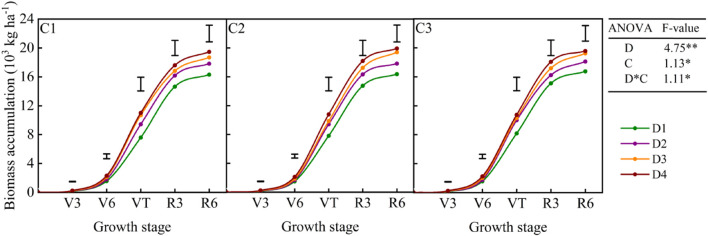
Aboveground biomass accumulation of dryland maize under different planting densities and cultivar treatments. D1: 52,500 plants ha^–1^; D2: 67,500 plants ha^–1^; D3: 82,500 plants ha^–1^; D4: 97,500 plants ha^–1^; C1: Yuyu22; C2: Zhengdan958; C3: Xianyu335; D, planting density; C, cultivar. Data represent the average values over the experimental period (2013–2018). Vertical bars represent the least significant difference (LSD) value at *p* < 0.05.

Maize biomass yield varied significantly with planting density and cultivar over the six cropping seasons (*p* < 0.05) (Table 1). Aboveground biomass accumulation increased with the increase in planting density (Figure 2), with the highest value recorded in the D4 treatment. Biomass yield accumulation increased slowly from the V3 to V6 stage and rapidly from the V6 to VT stage, with the highest value recorded at physiological maturity (Figure 2). In contrast, HI decreased with the increase in planting density (Table 1).

**TABLE 1:** Maize grain yield and its components in different treatments.

Factor		Kernel number per meter	Kernel weight (g 100 seed^–1^)	Grain yield (kg ha^–1^)	Biomass yield (kg ha^–1^)	HI (%)	PUE (kg ha^–1^ mm^–1^)
Density (D)	D1	2680c	28.1a	7592c	16592c	45.1a	24.8c
	D2	2993b	26.5b	8507a	18363b	46.1a	26.8a
	D3	3128a	25.7c	8126b	19785a	42.9b	25.5b
	D4	3150a	23.6d	7411c	20031a	36.1c	25.2bc
Cultivar (C)	C1	2827c	27.1a	7665b	18012b	42.3a	24.8b
	C2	3110b	25.9b	8095a	18556a	43.4a	26.2a
	C3	3186a	25.1b	8062a	18653a	42.8a	26.0a
**Source of variation**							
D		***	***	***	***	***	***
C		***	***	**	*	ns	***
D*C		*	*	*	**	**	***

D1: 52,500 plants ha^–1^; D2: 67,500 plants ha^–1^; D3: 82,500 plants ha^–1^; D4: 97,500 plants ha^–1^; C1: Yuyu22; C2: Zhengdan958; C3: Xianyu335; HI, harvest index; PUE, precipitation use efficiency. Data represent the average values over the experimental period (2013–2018). Different letters within the same treatment represent significant differences at p < 0.05 [least significant difference (LSD) test]. Asterisks indicate the significance level of the correlation (*p < 0.05, **p < 0.01, ***p < 0.001). ns, non-significant (p > 0.05).

Yield and its components were significantly affected by density and cultivar over the 6 years (*p* < 0.05) (Table 1). The average ear number per square meter increased, with the increase in planting density, whereas the 100-kernel weight decreased. Grain yield did not increase with the increase in planting density and showed the highest value in the D2 treatment (Table 1). The interaction between density and cultivar had significant effects on yield and its components (*p* < 0.05). The yield variation (CV) increased with the increase in planting density, but the SYI value decreased (Table 2). Differences in yield stability were detected among the three cultivars, and the C2 and C3 showed lower yield variation than the C1. These results indicate that high planting density raises the yield variability and decreases the yield sustainability of dryland maize, which was not conducive to the sustainable development of dryland farming.

**TABLE 2:** Yield stability index (CV, %) and sustainable yield index (SYI) of dryland maize in different treatments.

Density	Cultivar	Mean (kg ha^–1^)	SD	CV (%)	SYI
D1	C1	7,544	1,983	26.3	0.59
	C2	7,539	2,021	26.8	0.57
	C3	7,291	1,809	24.8	0.61
D2	C1	7,776	2,127	27.4	0.55
	C2	8,458	2,209	26.1	0.58
	C3	8,333	2,149	25.8	0.58
D3	C1	7,396	2,620	35.4	0.51
	C2	8,314	2,754	33.1	0.52
	C3	8,204	2,726	33.2	0.51
D4	C1	6,212	2,563	41.3	0.40
	C2	6,778	2,620	38.6	0.42
	C3	7,855	2,803	35.7	0.50

*D1: 52,500 plants ha^–1^; D2: 67,500 plants ha^–1^; D3: 82,500 plants ha^–1^; D4: 97,500 plants ha^–1^; C1: Yuyu22; C2: Zhengdan958; C3: Xianyu335. SD, standard deviation; CV, coefficient of variation.*

### Canopy Structural Characteristics

#### Dynamics of Leaf Area Development

**FIGURE 3:**
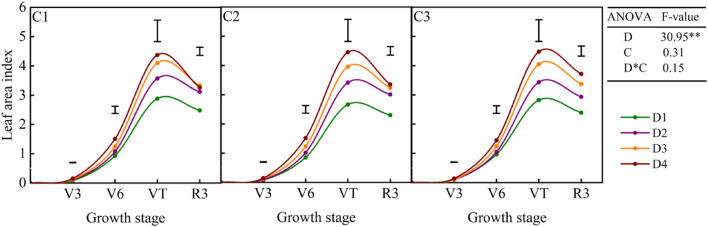
Dynamics of the leaf area index (LAI) of dryland maize under different planting densities and cultivar treatments. D1: 52,500 plants ha^–1^; D2: 67,500 plants ha^–1^; D3: 82,500 plants ha^–1^; D4: 97,500 plants ha^–1^; C1: Yuyu22; C2: Zhengdan958; C3: Xianyu335; D, planting density; C, cultivar. Data represent the average values over the experimental period (2013–2018). Vertical bars represent the LSD value at *p* < 0.05.

The average value of LAI over the six cropping seasons increased with the increase in planting density (Figure 3), with the highest value recorded in the D4 treatment for all cultivars. The average LAI values for D2, D3, and D4 treatments were 19–27%, 38–44%, and 45–60%, respectively, higher than that in the D1 treatment. In all treatments, LAI increased slowly from the V3 to V6 stage before increasing rapidly from the V6 to VT stage, peaking at the VT stage, and then decreasing gradually. However, the amplitude of decline varied among the three cultivars, with the most rapid decline detected in the C1 (Figure 3).

#### Intercepted Photosynthetically Active Radiation

The IPAR captured by maize canopy was significantly affected by planting density and cultivar over the six cropping seasons (*p* < 0.05) (Table 3). Compared with the D1 treatment, the IPAR values increased by 13.5, 18.6, and 23.7% in the D2, D3, and D4 treatments, respectively. The IPAR values of the C2 and C3 were 9.3 and 8.2%, respectively, lower than that of the C1.

**TABLE 3:** Intercepted photosynthetically active radiation (IPAR) and radiation use efficiency (RUE) of dryland maize in different treatments.

Factor		IPAR (MJ m^–2^)	RUE_GY_ (g MJ^–1^)	RUE_BY_ (g MJ^–1^)
Density (D)	D1	877d	0.86a	1.89a
	D2	995c	0.85a	1.84b
	D3	1040b	0.77b	1.81b
	D4	1085a	0.67c	1.80b
Cultivar (C)	C1	1001a	0.77b	1.80b
	C2	908b	0.82a	1.87a
	C3	919b	0.80a	1.87a
**Source of variation**				
D		***	***	*
C		*	**	**
D*C		ns	*	ns

D1: 52,500 plants ha^–1^; D2: 67,500 plants ha^–1^; D3: 82,500 plants ha^–1^; D4: 97,500 plants ha^–1^; C1: Yuyu22; C2: Zhengdan958; C3: Xianyu335; RUE_GY_, radiation use efficiency of grain yield; RUE_BY_, radiation use efficiency of biomass yield. Data represent the average values over the experimental period (2013–2018). Different letters following means in different treatments represent significant differences at p < 0.05 (LSD test). Asterisks indicate the significance level of the correlation (*p < 0.05, **p < 0.01, ***p < 0.001). ns, non-significant (p > 0.05).

#### Photosynthetic Characteristics and Chlorophyll Content

**FIGURE 4:**
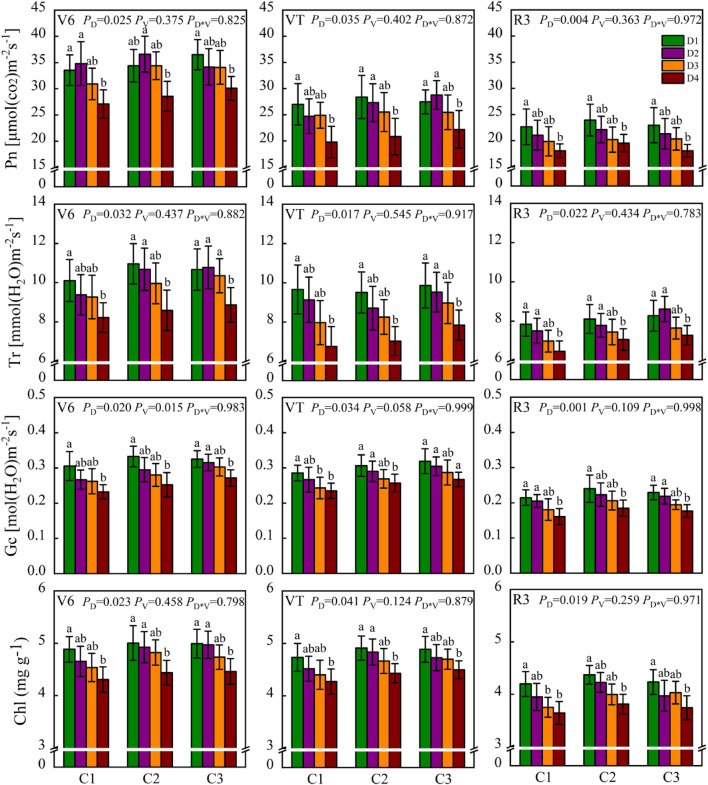
Net photosynthetic rate (Pn), transpiration rate (Tr), stomatal conductance (Gc), and leaf chlorophyll content (Chl) of dryland maize under different treatments. D1: 52,500 plants ha^–1^; D2: 67,500 plants ha^–1^; D3: 82,500 plants ha^–1^; D4: 97,500 plants ha^–1^; C1: Yuyu22; C2: Zhengdan958; C3: Xianyu335. Data represent mean ± SD over six cropping seasons. *P*-values of the ANOVA of density (*P*_D_), cultivar (*P*_V_), and their interaction (*P*_D__*__V_) were also shown.

The photosynthetic characteristics of dryland maize were significantly affected by planting density over the 6 years (*p* < 0.05) (Figure 4). Compared with the D1 treatment, the Pn in D2, D3, and D4 treatments, respectively, decreased by an average of 1.2, 4.8, and 17.8% at the V6 stage, by 2.4, 8.4, and 24.1% at the VT stage, and by 7.3, 13.1, and 19.9% at the R3 stage. Similar trends were observed for Tr and Gc. The leaf chlorophyll content of D2, D3, and D4 treatments also decreased by 2.2–5.1%, 5.3–8.0%, and 9.1–12.5% (Figure 4), respectively, compared with the D1 treatment. However, no significant differences in photosynthetic characteristics and chlorophyll content were observed among the different maize cultivars in most years.

### Resource Use Efficiency

#### Precipitation Use Efficiency

The PUE of maize was significantly affected by planting density and cultivar over the six cropping seasons (*p* < 0.05) (Table 1). Similar to the trend shown by grain yield, PUE decreased with the increase in planting density, reaching the highest level in the D2 treatment. Compared with the D2 treatment, the PUE of D3 and D4 treatments decreased by 4.9 and 6.0%, respectively. The interaction between planting density and cultivar had no significant effect on the PUE over the six cropping seasons.

#### Radiation Use Efficiency

The RUE of maize was significantly affected by planting density and cultivar among the six cropping seasons (*p* < 0.05) (Table 3). Although IPAR increased with the increase in planting density, the RUE showed the opposite trend (Table 3). Compared with the D2 treatment, the RUE of D3 and D4 treatments decreased by 9.4 and 21.2%, respectively, for grain yield and by 1.6 and 2.2%, respectively, for biomass yield. The interaction between planting density and cultivar had a significant effect on RUE for grain yield (*p* < 0.05).

#### Nitrogen Uptake and Utilization

The NUPE and NUE were significantly affected by planting density over the 6 years (*p* < 0.05) (Table 4). Total nitrogen uptake and nitrogen uptake for grain yield did not increase with the increasing planting density and reached the highest values in the D2 treatment. NHI decreased with the increase in planting density, indicating reduced translocation of nitrogen from vegetative organs to grains. Compared with the D1 treatment, the D2, D3, and D4 treatments showed an increase in NUE, NUPE, and NPE by −1.3 to 5.8%, −3.7 to 5.6%, and −2.2 to 12.2%, respectively. However, only NUPE and NPE showed significant differences among the three cultivars, and the C2 and C3 showed higher yields than the C1 (Table 4). The interaction between planting density and cultivar was significant for NPE (*p* < 0.05).

**TABLE 4:** Nitrogen uptake and utilization by dryland maize in different treatments.

Factor		Nitrogen uptake (kg ha^–1^)		NHI (%)	NUE (kg kg^–1^)	NUPE (kg kg^–1^)	NPE (kg kg^–1^)
		Grain	Total				
Density	D1	74.0ab	121.1ab	60.4ab	62.6b	0.54ab	33.7c
(D)	D2	81.3a	128.6a	61.8a	66.1a	0.57a	37.8a
	D3	76.5ab	121.0ab	61.2a	66.4a	0.54ab	36.1b
	D4	71.5b	117.9b	58.8b	61.7b	0.52b	32.9c
Cultivar	C1	71.6b	115.3b	60.9a	65.2a	0.51b	34.1b
(C)	C2	77.6a	123.9a	61.2a	64.6a	0.55a	36.0a
	C3	79.5a	129.2a	60.7a	63.7a	0.57a	35.8a
Source of variation							
D		*	*	*	*	*	***
C		*	*	ns	ns	*	**
D*C		ns	ns	ns	ns	Ns	**

D1: 52,500 plants ha^–1^; D2: 67,500 plants ha^–1^; D3: 82,500 plants ha^–1^; D4: 97,500 plants ha^–1^; C1: Yuyu22; C2: Zhengdan958; C3: Xianyu335; NHI, nitrogen harvest index; NUE, nitrogen use efficiency; NUPE, nitrogen uptake efficiency; NPE, nitrogen productive efficiency. Data represent the average values over the experimental period (2013–2018). Different letters within the same treatment represent significant differences at p < 0.05 (LSD test). Asterisks indicate the significance level of the correlation (*p < 0.05, **p < 0.01, ***p < 0.001). ns, non-significant (p > 0.05).

#### Relationships Among Yield, Harvest Index, Nitrogen Uptake Efficiency, Nitrogen Productive Efficiency, Precipitation Use Efficiency, and Radiation Use Efficiency

**FIGURE 5:**
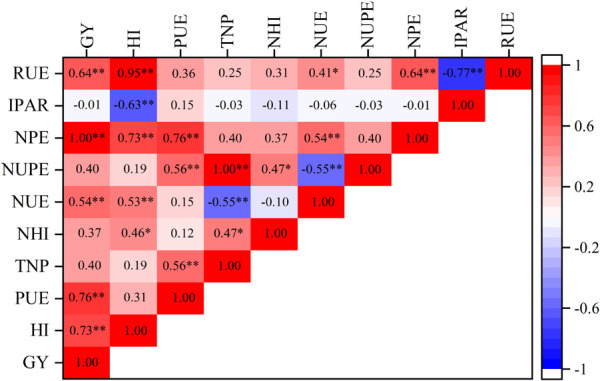
Correlation coefficients of maize yield and resource use efficiency. GY, grain yield; HI, harvest index; PUE, precipitation use efficiency; TNP, total nitrogen uptake; NHI, nitrogen harvest index; NUE, nitrogen use efficiency; NUPE, nitrogen uptake efficiency; NPE, nitrogen productive efficiency; IPAR, intercepted photosynthetically active radiation; RUE, radiation use efficiency. Asterisks indicate the significance level of the correlation (^∗^*p <* 0.05, ^∗∗^*p <* 0.01, ^∗∗∗^*p <* 0.001). ns, non-significant (*p >* 0.05).

The relationships between maize grain yield and HI and those of NUPE with NUE, NPE, and RUE are shown in Figure 5. Grain yield was significantly positively correlated with HI, PUE, NUE, NPE, and RUE, but it showed no significant correlation with total nitrogen uptake and NHI. These correlations suggest that maize productivity under high planting density is limited by the relatively low translocation of assimilates from vegetative organs to grains, resulting in low resource use efficiency and relatively low productivity.

## Discussion

### Canopy Structure and Photosynthetic Characteristics

Previous research has demonstrated that increasing planting density improves maize canopy closure, i.e., rapid canopy establishment and leaf area expansion, leading to greater IPAR, which contributes to greater radiation capture (Teixeira et al., 2014; Du et al., 2021). Similar results were obtained in this study. Compared with the D1 treatment, the average LAI values of the three cultivars increased by 19–27%, 38–44%, and 45–60% in D2, D3, and D4 treatments, respectively. After the VT stage, the LAI value decreased due to the shedding and senescence of plant leaves, but the amplitude of this decline was small in the low density, which is beneficial to the assimilating of photosynthetic products and resulting in higher partitioning of carbohydrates to the ear. This was mainly related to the lower interplant competition between plants, which has been reported in maize (Hammer et al., 2009; Rossini et al., 2011). Additionally, low-density crops maintain high green leaf area and leaf chlorophyll content and were accompanied by higher photosynthetic characteristics, such as Pn and Gc (Figure 4). The Gc affects the exchange of CO_2_ and H_2_O between leaves and the environment, as an adaptive mechanism to cope with drought stress (Hernández et al., 2020). Zhu et al. (2010) showed that photosynthetic efficiency is closely related to the regulation of stomatal opening and leaf chlorophyll content, and the increase in crop productivity relies on improved photosynthesis. Thus, optimizing canopy structure and maintaining photosynthetic capacity while increasing the resource use efficiency would be the key to improve the maize yield by optimizing planting density. One limitation of this study is that we monitored the photosynthetic characteristics and chlorophyll content of only the ear leaves, and the photosynthetic performance of the whole maize population remains unknown. Further investigation will help explain yield formation from the perspective of group light energy efficiency.

### Grain Yield

In this study, biomass yield increased with the increase in plant density, whereas grain yield showed a parabolic relation with planting density (Table 1). The increasing of planting density results in lower light intensity in the canopy, but a certain grain yield needs more leaf area (to realize a high canopy photosynthesis rate) to support its grain filling and crop yield (Du et al., 2021). Thus, the HI decreased dramatically with increasing planting density. Increasing planting density significantly improved LAI and IPAR of the canopy, eventually resulting in a significant increase in aboveground dry matter accumulation (Teixeira et al., 2014). However, as planting density increased, the photosynthetic characteristics of plants declined, resulting in lower crop photosynthetic assimilation and productivity per plant, which might explain the decrease in maize yield observed in this study at high planting density. These results indicate that dryland maize productivity at high planting density is limited by the relatively low translocation of assimilates to grains. Therefore, pursuing high planting density is not a desirable strategy in the rainfed farming system, while the relatively lower planting density may be more conducive to the effective use of limited resources of semiarid environments. Increasing planting density also increased the yield variability (CV, %) and decreased the yield sustainability of dryland maize (Table 2). Mylonas et al. (2020) also revealed that CV (%) values of plant yield increased when planting density increased, mainly due to increased competition for resources, especially for soil water in rainfall agroecosystems. While under lower planting density, the available water per plant increases, which can maintain the growth of crops and filling of grain. The cultivar is another factor affecting grain yield response to density and stability, as shown by previous studies (Berzsenyi and Tokatlidis, 2012; Chen et al., 2017; Solomon et al., 2017), as well as our current results. In this study, C2 and C3 showed higher yield and yield stability over the six cropping seasons than the C1 (Table 2). The lower yield of the C1 was associated with the rapid decline in LAI after the tasseling stage (Figure 3). This is consistent with previous findings reported that the reduction in green LAI results decreases the fraction of total radiation intercepted and leads to lower carbohydrate remobilization from leaves to the ear (Xue et al., 2010).

### Resource Use Efficiency

Improving the resource use efficiency of crop plants is the main strategy to realize the sustainable development of agriculture. In this study, PUE was significantly affected by planting density and cultivar (*p* < 0.05) (Table 1). A similar trend was shown by grain yield, and PUE did not increase with the increase in planting density but showed a parabolic relation with planting density. Results by Tokatlidis et al. (2011) and Berzsenyi and Tokatlidis (2012) highlighted the importance of maize cultivars that are less dependent on high planting density to increase resource use efficiency in non-irrigated land. Although increments in IPAR were in accordance with increasing LAI, they did not promote higher RUE. This is partly because light attenuation within the canopy was increased under higher plant population due to shading, and relatively more light captured by the upper canopy has been suggested to reduce the whole plant photosynthetic efficiency, which in turn decreases the RUE (Du et al., 2021). In addition to water and radiation, crop productivity also depends on the absorption of nutrients and allocation of assimilates (Teixeira et al., 2014; Xu et al., 2017; Zhang et al., 2019a). In this study, increasing plant population did not increase the NUPE and NUE over 6 years (Table 4). This was mainly because increasing planting density decreases the capacity of the crop to accumulate nitrogen per unit green LAI (Ciampitti and Vyn, 2011), thus decreasing the NPE. Therefore, provided cultivars have high plant yield efficiency, and using lower planting density to enhance crop resilience to extremely fluctuating environments will be more meaningful for the long-term development of dryland agriculture. Differences in cultivar characteristics are one of the main reasons for the differences in resource use efficiency (i.e., radiation, water, and nutrients). Density-tolerant cultivars (C2 and C3) exhibited higher resource use efficiency than the C1 under the same climatic conditions. This was mainly related to the light distribution through the canopy, which was increased for density-tolerant cultivars due to their upright leaves and small leaf angles (Xue et al., 2010). This resulted in relatively more light being captured by the lower canopy of density-tolerant cultivars, thus improving their resource use efficiency.

## Conclusion

This study evaluated the effects of maize planting density and cultivar on canopy structure, photosynthetic traits, yield, and resource use efficiency. The increase in planting density improved the LAI and canopy closure and consequently enhanced the capacity of maize plants to uptake nutrients, absorb soil water, and capture PAR, leading to higher crop productivity. However, increased planting density decreased the photosynthetic characteristics (Pn and Gc) and leaf chlorophyll content, which resulted in lower photosynthetic capacity. These alterations constitute the key mechanisms underlying the decline in yield and resource use efficiency at high planting density. These results suggest that high planting density reduces maize yields mainly through a decline in photosynthetic efficiency and conversion efficiency, which translates into a proportional reduction in resource use efficiency. Therefore, optimizing planting density *via* improved high plant yield efficiency and resource use efficiency to enhance yield stability will be more beneficial to the long-term development of dryland agriculture. Different cultivars also show different responses to planting density; C2 and C3 showed better canopy structure, yield stability, and resource use efficiency than C1. Different cultivars also show different responses to planting density regarding canopy structure, yield stability, and resource use efficiency. Provided of high plant yield efficiency, cultivation of density-tolerant cultivars with a reasonable decrease in planting density can increase maize yield stability and resource use efficiency in rainfed agroecosystems, thus facilitating the development of sustainable agriculture.

## Data Availability Statement

The original contributions presented in the study are included in the article/supplementary material, further inquiries can be directed to the corresponding authors.

## Author Contributions

JL conceived and designed the experiments. YZ and ZX performed the experiments. YZ and RW analyzed the data and wrote the manuscript. YZ, JL, and RW reviewed and revised the manuscript and corrected the English language. All authors reviewed and approved the manuscript for publication.

## Conflict of Interest

The authors declare that the research was conducted in the absence of any commercial or financial relationships that could be construed as a potential conflict of interest.

## Publisher’s Note

All claims expressed in this article are solely those of the authors and do not necessarily represent those of their affiliated organizations, or those of the publisher, the editors and the reviewers. Any product that may be evaluated in this article, or claim that may be made by its manufacturer, is not guaranteed or endorsed by the publisher.

## Footnotes

1
http://data.cma.cn/
